# Mindful breath awareness meditation facilitates efficiency gains in brain networks: A steady-state visually evoked potentials study

**DOI:** 10.1038/s41598-018-32046-5

**Published:** 2018-09-12

**Authors:** Benjamin Schöne, Thomas Gruber, Sebastian Graetz, Martin Bernhof, Peter Malinowski

**Affiliations:** 1 Institute of Psychology, Experimental Psychology I, Osnabrück University, Osnabrück, Germany; 2 Research Centre for Brain and Behaviour, Liverpool John Moores University, Liverpool, UK

## Abstract

The beneficial effects of mindfulness-based therapeutic interventions have stimulated a rapidly growing body of scientific research into underlying psychological processes. Resulting evidence indicates that engaging with mindfulness meditation is associated with increased performance on a range of cognitive tasks. However, the mechanisms promoting these improvements require further investigation. We studied changes in behavioural performance of 34 participants during a multiple object tracking (MOT) task that taps core cognitive processes, namely sustained selective visual attention and spatial working memory. Concurrently, we recorded the steady-state visually evoked potential (SSVEP), an EEG signal elicited by the continuously flickering moving objects, and indicator of attentional engagement. Participants were tested before and after practicing eight weeks of mindful breath awareness meditation or progressive muscle relaxation as active control condition. The meditation group improved their MOT-performance and exhibited a reduction of SSVEP amplitudes, whereas no such changes were observed in the relaxation group. Neither group changed in self-reported positive affect and mindfulness, while a marginal increase in negative affect was observed in the mindfulness group. This novel way of combining MOT and SSVEP provides the important insight that mindful breath awareness meditation may lead to refinements of attention networks, enabling more efficient use of attentional resources.

## Introduction

Over the last decades, research investigating the effects of mindfulness-based interventions (MBIs) has grown exponentially^1–7^. More recently, attention has been directed towards understanding the neuro-cognitive processes that contribute to the therapeutic effects of MBIs and the meditation practices included in these MBIs. Emerging evidence suggests that meditation training or the participation in MBIs can result in improvements of cognitive processes such as attentional functions^8,9^, working memory^10^, and executive and meta-cognitive functions^11^. Such improvements in cognition are thought to interact with concurrent refinements of emotion regulation skills, resulting in enhanced psychological functioning and wellbeing^12–14^.

Although numerous researchers are dedicated to unravelling the functional and structural changes associated with mindfulness training, the understanding of the underlying psychological and neural mechanisms is currently limited^15,16^. To address this, several limitations have to be overcome, in particular the reliance on cross-sectional rather than longitudinal designs and the lack of active control conditions. Furthermore, the use of complex MBIs that include various components other than meditation hampers the ability to isolate specific effects that can be attributed directly to mindfulness meditation practice. For instance, prominent MBIs such as Mindfulness-based Stress Reduction (MBSR)^17^, Mindfulness-based Cognitive Therapy (MBCT)^18^ or Mindfulness-based Relapse Prevention (MBRP)^19^ include a whole range of components other than meditation such as psycho-education, yoga exercises, stretching and group discussions, while also integrating several different meditation exercises. As a result, it is impossible to gain certainty if, and to what extent, observed effects of these MBIs can be attributed to meditation in general, or to a specific meditation exercise in particular^15,20,21^. A large-scale MBCT dismantling trial found that an intervention identical to MBCT but excluding all meditation exercises, was therapeutically as effective as was standard MBCT with meditation practice^22^. As the field matures and such results are coming forward it is increasingly recognized, that it is essential to focus the research on the effects of *specific* meditation exercises and to describe these exercises clearly rather than getting trapped in the ambiguities of relying exclusively on “mindfulness” as umbrella term^16,23^.

Despite concerns regarding specific definitions of mindfulness, there is general agreement that mindfulness meditation as considered within psychological contexts entails paying attention to experiences that arise in the present moment combined with maintaining a non-judging, open, and accepting attitude^13,17,24^ while the role of attentional stability and associated meta-cognition as a foundational feature is highlighted^9,15,25^. In line with this, several studies confirmed that engaging in mindfulness meditation results in more efficient use of attentional control functions across a range of cognitive tasks^9,26,27^.

It is intriguing that a mental exercise that “merely” entails the voluntary focus on a simple object, such as the sensation of one’s own breath, combined with a non-reactive and accepting awareness of concurrently arising mental phenomena, can have far-reaching effects on cognitive functions. Such improvements have been explained in terms of brain network training, which is thought to enhance the functioning of interacting brain networks of attention^21,25,28^. For instance, each sequence of detecting that the mind got entangled in distractions such as mind wandering would engage the salience, executive control and orienting networks^25,28^ and over time lead to efficiency gains of these networks.

Such efficiency gains have been observed in different cognitive tasks that probe the functioning of these networks. For example, 16 weeks of regular, brief mindful breath awareness meditation enhanced the N2 event-related potential (ERP) during a computerised Stroop task, indicating improved attention allocation to the colour word stimuli. This improvement was associated with a reduction in the P3 ERP, signifying more efficient – or less resource-intensive – processing of incongruent stimuli that elicited a response conflict^29^. Similarly, several studies using the attentional blink task demonstrated more efficient allocation of attentional resources over time as a result of engaging with meditation practice^30–32^. Other studies have shown improved attentional functions and reduced engagement with distracting stimuli *during* meditation^33–35^, providing a plausible indication that attentional engagement during meditation transfers to generalised improvements of attentional functions.

Furthermore, engaging in meditation involves working memory functions, for example, while keeping the meditation object or the specific meditation instructions actively in mind. In line with this, research has demonstrated improvements in working memory, the capacity to retain and manipulate goal-relevant information, as a result of meditation practice^10,36^. The ability to sustain the meditation object in working memory and to return to it by rapidly recognising distraction and disengaging from it are thus key cognitive processes involved in mindfulness meditation practice.

Interestingly, evidence from cognitive neuroscience demonstrates the close interplay between attention and working memory, highlighting the important role of selective attention in encoding information in working memory^37,38^. In addition, the efficiency of allocating attentional resources to goal-relevant rather than irrelevant, distracting information predicts working memory performance^39^. Fukuda and Vogel^40^ demonstrate that the ability to rapidly disengage from distracting information is an important contributor to high-capacity working memory performance.

Multiple object tracking (MOT) paradigms, which combine sustained attention and visual working memory demands, have been employed successfully to investigate cognitive performance under challenging conditions^41,42^. Such research demonstrated that video gaming can lead to improved MOT performance^43^ and that, compared to matched controls, radar operators demonstrate superior performance on that task^44^. Furthermore, MOT appears to be a useful tool for tracking the development of visual attention skills in children^45,46^, for revealing age-related attentional decline^47,48^, and for investigation reductions of brain functional connectivity during high cognitive demand^49^. Störmer, Winther, Li, and Andersen^50^ combined MOT with electrophysiological recording of steady-state visual evoked potentials (SSVEP) elicited by flickering moving objects and confirmed that the continuous selective attentional enhancement of the tracked objects is directly associated with performance.

The SSVEP is the oscillatory response of cortical networks to flickering stimuli with the same fundamental frequency^51^. Its amplitude is an indicator for the allocation of selective attention and reflects the amount of neural resources devoted to the perception of an exogenous stimulus, but also to subsequent endogenous steps of processing^52^. The SSVEP initially originates in the primary visual cortices and then spreads along the neural pathways to higher areas, which are associated with relevant cognitive operations^53,54^.

Although the SSVEP is a particularly powerful tool for tracking the continuous allocation of attention over time, it has not yet been used for investigating attentional processes related to meditation. Similarly, the MOT has only rarely been used to study meditation. Despite its sensitivity to expertise and to some forms of training, and although it engages selective attention and working memory, to our knowledge only one study used a MOT paradigm to investigate attention and working memory in relation to meditation and mindfulness. Hartkamp and Thornton^55^ reported no improvements in tracking performance after a 6-day meditation retreat. However, methodological limitations (such as non-matched control group) and lacking information regarding participants’ initial meditation expertise makes it difficult to appraise these results.

In the current study, we aimed to combine the advantages of MOT and SSVEP to investigate whether eight weeks of mindful breath awareness meditation leads to improved neural network efficiency of sustained visual attention during encoding and maintenance of information in visual short-term memory. To achieve this, we employed a MOT task while concurrently recording SSVEPs. Importantly, we included an active control group, in which participants underwent training in progressive muscle relaxation (PMR)^56^, an approach that is sufficiently similar to mindfulness meditation while at the same time not including directions regarding the two key features of mindfulness meditation, namely the development of attentional stability and the emphasis on a non-judging, accepting attitude towards all experience. To allow a straightforward interpretation of meditation effects, participants in the meditation group engaged in only one exercise, mindful breath awareness meditation (MED), rather than a typical multi-component MBI programme.

The MOT task required participants to track simultaneously between two and five independently moving targets embedded in a total of 15 moving objects that are identical to the target. With increasing number of targets task difficulty increases significantly, avoiding potential ceiling effects that may have contributed to some ambiguous findings in this field^25^. The SSVEP was elicited by the continuous flickering of all moving objects and was used as an index of engagement and efficiency of attention networks.

## Results

### Practice

The meditation group practiced on average for 41.71 (SD = 22.02) minutes per week in 3.86 meditation sessions. The PMR group acquired a similar amount of practice hours. Participants exercised for on average 42.41 (SD = 17.71) minutes per week in 2.99 sessions, The groups did not differ in the amount (*t*(31) = −0.10, *p* = 0.921) or frequency (*t*(31) = 1.97, *p* = 0.058) of weekly practice.

### Questionnaires

#### PANAS

For self-reported positive affect (PANAS-PA), we found a significant effect for factor TIME *F*(1,27) = 4.78, *p* < 0.05, η² = 0.15, but no significant interaction of TIME and GROUP, *F*(1,27) = 1.83, *p* = 0.187; η² = 0.063. For negative affect (PANAS-NA), we found a significant effect for factor TIME *F*(1,26) = 5.17, *p* < 0.05; η² = 0.166, which was further qualified by a marginally significant interaction of TIME and GROUP, *F*(1,26) = 7.16; *p* < 0.06, η² = 0.216. The groups did not differ in negative affect prior to the training (MED: M = 11.8, SD = 1.38; PMR: M = 11.47; SD = 1.13, *t*(29) = 0.76; *p* = 0.45), while after the training the meditation group reported higher negative affect than the PMR group (MED: *M* = 14.27; SD = 3.90; PRM: M = 11.12, SD = 1.41, *t*(17.39) = 2.945; *p* < 0.001). This difference emerged due to an increase in negative affect within the meditation group, PRE: *M* = 11.81; SD = 1.38; POST: *M* = 14.27; SD = 3.90, *t*(13) = −2.65, *p* < 0.05, but should be interpreted with care as the associated TIME × GROUP interaction was only marginally significant.

#### FFMQ

The ANOVA for self-reported mindfulness (FFMQ) exhibited no significant main effect for TIME, *F*(1,31) = 1.17, *p* = 0.29, η² = 0.036 or GROUP, F(1,31) = 0.012, p = 0.731, η² = 0.004, and no interaction between TIME and GROUP, *F*(1,31) = 0.05, *p* = 0.82, η² = 0.002, showing that the total FFMQ score was not affected by the training.

### MOT-Task

Concerning response accuracy, we found a significant main effect for TIME, *F*(1,32) = 4.96; *p* < 0.05; η² = 0.13, which is further qualified by a significant interaction between TIME and GROUP, *F*(1,32) = 4.30; *p* < 0.05; η² = 0.12. As expected, we also found a main effect for factor CONDITION, *F*(2.60, 83.10) = 112.81; *p* < 0.001; η² = 0.78. Comparing conditions with increasing difficulty (2 vs 3 targets, 3 vs 4 targets, 4 vs 5 targets) confirmed that with increasing numbers of target objects the accuracy declined (all t(33) > 2.27; all p ≤ 0.03). Accuracy for 2 targets was 84%, for 3 targets 69%, for 4 targets 66%, and for 5 targets 61%. Because CONDITION did not interact with any of the other factors, for subsequent analyses all data were averaged across CONDITION.

**Figure 1:**
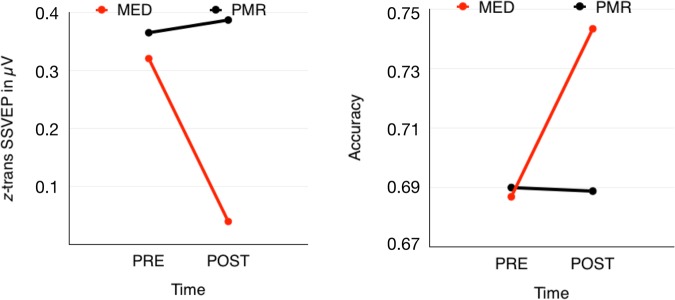
(**a**) Results for the SSVEP in the 500–6800 ms time window showing a reduction of amplitude only within the mindfulness training group. This deflection is accompanied by (**b**) enhanced performance in the multiple-object-tracking task.

A post-hoc t-test that explored the TIME × GROUP interaction showed no difference with respect to performance in the MOT task before the training, *t*(32) = 0.12; *p* = 0.90. However, after the intervention the groups differed significantly, *t*(32) = 2.08; *p* < 0.05, resulting from an increase in accuracy within the meditation group, t(16) = −2.67; *p* < 0.05, with no significant change in the progressive muscle relaxation group, *p* = 0.89 (Fig. 1b).

### Electrophysiological data

Within the three selected time windows as well as over the entire time the results of electrophysiological measurement principally resemble the MOT performance pattern. The analysis of SSVEP data from 500 ms to 6800 ms after movement onset revealed a marginally significant main effect for factor TIME, *F*(1,32) = 4.26; *p* = 0.057; η² = 0.11, as well as a significant interaction between the factor TIME and GROUP, *F*(1,32) = 5.30; *p* < 0.05; η² = 0.14. We found no further effects. Consequently, also for the SSVEPs we averaged over the factor CONDITION. Probing the TIME × GROUP interaction no significant difference between the MED- and the PMR-group was observed before participants engaged in the training programs (*M*_Med_ = 0.32, SD_MED_ = 0.33, *M*_PMR_ = 0.36, SD_PMR_ = 0.39), *t*(32) = 0.36; *p* = 0.72. Furthermore, the PMR training did not modulate the SSVEP amplitudes in any way. However, within the MED group SSVEP-amplitudes were significantly reduced after the training, *t*(16) = 2.77; *p* < 0.05, and considerably smaller than the amplitudes of the PMR group after training (*M*_Med_ = 0.04, SD_MED_ = 0.41, *M*_PMR_ = 0.39, SD_PMR_ = 0.31), *t*(32) = 2.77; *p* < 0.01 (Fig. 1a).

In time window T1 (500 –2000 ms) we found a main effect for factor TIME, *F*(1,32) = 4.21; *p* < 0.05; η² = 0.12, as well as significant TIME × GROUP interaction, *F*(1,32) = 5.53; *p* < 0.05; η² = 0.15. No further effects were found (*p* > 0.20). A post-hoc *t-*test showed no differences before training, *t*(32) = 0.2; *p* = 0.84. After training amplitudes were lower in the MED group than the PMR group, *t*(16) = 3.07; *p* < 0.01). No significant changes were found within the PMR group (*p* > 0.80). The averaged SSVEP amplitude of the MED group was again considerably diminished compared to the PMR group in the post measurement. In the following two time windows T2 (2000 –4750 ms) and T3 (4750 –6800 ms) the main effect for TIME turned out to be marginally significant in the first time window, *F*(1,32) = 4.07; *p* = 0.052; η² = 0.11, but not in the second one, *F*(1,32) = 1.72; *p* = 0.2, η² = 0.05. However, in both time windows the interaction between TIME and GROUP was significant [T2: *F*(1,32) = 4.17; *p* < 0.05; η² = 0.15] or marginally significant [T3: *F*(1,32) = 3.87; *p* = 0.058; η² = 0.11], with no further significant effects. Post-hoc t-tests exhibit the familiar pattern. No significant differences were present before the training, and meditation is associated with a reduction of SSVEP amplitudes [T2: *t*(16) = 2.58; *p* < 0.05; T3: t(16) = 2.16; p < 0.05], whereas PMR did not lead to SSVEP changes. In both time windows the SSVEP amplitudes after training are smaller in the MED group than in the PMR group, *t*(32) = 2.89; *p* = 0.01 and t(32) = 2.52; *p* < 0.05, respectively.

## Discussion

The present study examined the effects of 8 weeks regular brief mindful breath awareness meditation on neural processes involved in a sustained visual attention and short-term memory task. Specifically, we employed the MOT task to investigate the selection, encoding and maintenance of task-relevant information in visual short-term memory in the presence of competing distractors. In the meditation group we found training-related improvements in MOT performance combined with a reduction of the SSVEP, whereas no such changes were observed in the progressive muscle relaxation group. These meditation-specific changes were not accompanied by improved self-reported affect and a merely marginally significant increase in negative affect. Also, self-reported mindfulness did not change significantly.

The use of an active control group that engaged for the same amount of time as the meditation group in relatively similar activities, but did not exhibit changes in MOT performance or SSVEP amplitudes, allows us to conclude that the observed changes truly result from meditation practice. Furthermore, focusing on *one* particular meditation exercise, rather than a conglomerate of different practices as is the case in standard MBIs, provides information about the specific effects of this particular practice. Participants engaged in mindful breath awareness meditation, one of the most widely used basic form of meditation. It engages key components that are considered central to mindfulness meditation exercises, attentional stability, combined with non-judging, open and accepting awareness^24,25^. The mindful breath awareness meditation and the progressive muscle relaxation exercises incorporate attention to somatosensory experiences, either by focusing on sensations of various body parts (PMR) or on the sensation of the breath at nostrils or abdomen (MED). Whereas the primary aim of progressive muscle relaxation is to reduce stress by paying attention to the sensation of muscle relaxation, the mindfulness training explicitly emphasises attentional stability combined with an accepting, non-judging and non-engaging stance towards all experience arising the practice. Thus, while PMR and MED are similar in terms of the general structure of the delivery, they differ in terms of the specific instructions: The PMR group was instructed to manipulate their muscles and focus on the experience of relaxation, implying the value-judgment that this experience is positive. The mindfulness meditation instruction, on the other hand, emphasised not to manipulate the object of meditation (the breath), while remaining open and accepting to other arising experiences, such as thoughts, feelings, sounds, or bodily sensations. This meditation exercise incorporates executive control mechanisms, in particular maintaining attention to relevant stimuli, inhibition of automatic behaviour, and disengagement from ongoing involuntary internal or external distraction^9,11,25,57^. These two components have also been discussed in terms of focused attention (FA) and open monitoring (OM)^9^. Whereas FA relates to the facet of establishing and sustaining the attentional focus during meditation, OM denotes the open, curious and accepting mental attitude with which all arising mental phenomena are encountered. Because all our participants were novice meditators, we would expect that initially the main emphasis of their practice will have been on developing attentional stability (FA), while OM will have been developed as response to mind-wandering.

The MOT task is a highly demanding task and performance is unlikely to suffer from ceiling effects that may have affected previous studies of mindful breath awareness practice^29^. Successful engagement with the task requires selective visual attention, visual short-term memory and executive functions. The magnitude of the SSVEP is thought to be proportional to the amount of cortical resources that are allocated to perform a task^58^. In consequence, amplitude reductions would indicate that neuronal resources are utilised in a more efficient way. Thus, the meditation-specific pattern of improved MOT performance in conjunction with reduced SSVEP amplitudes is likely to result from an increased ability to ignore irrelevant distractors or to quickly disengage from them, preserving only relevant items in visual short-term memory^39^. It is likely to reflect efficiency gains that results from the training of interacting brain networks of attention during meditation which transfers to generalised improvements of attentional functions without meditation^21,25,59^.

This is the most recent of three studies that investigated the specific effects of the same mindful breath awareness meditation^29,60^. All three studies focused on and found improvements in specific cognitive functions as a result of engaging in meditation practice. However, in all three studies effects on self-reported mindfulness, wellbeing or affect were not clear-cut. Although in Moore *et al*.^29^ the meditation group improved in mindfulness (FFMQ) over 16 weeks of meditation practice, their mindfulness score after the training was equal to the score of the control group and specific improvements may have resulted from subdued pre-test scores. Similarly, mental wellbeing scores and total mindfulness scores (FFMQ) did not improve as a result of 8 weeks of meditation in the study by Malinowski *et al*.^60^. Furthermore, that study employed a modified Stroop task, which also tapped emotion processing, and did not find meditation-specific improvements of emotion processing. Here, we report the third study that did not find clear effects of mindful breath awareness practice on mindfulness, emotional processing or affect.

For explaining the lack of effects on these measures it is worth considering that self-report measures in longitudinal meditation studies need to be interpreted with particular care. Because mindfulness meditation, per se, aims at changing the way how participants relate to their internal experiences, including their emotional states, it is highly likely that the same questionnaire items are interpreted in qualitatively different ways before and after having been introduced to and having practiced mindfulness meditation. As discussed in more detail elsewhere^21^ to report on one’s own mental states requires a certain degree of mindfulness. As mindfulness is assumed to increase as a result of meditation practice, once own ability to be mindful may be evaluated in different ways. Moreover, the fact that meditation fosters a non-judgemental awareness of one’s own affective states, renders the comparison of self-reported affect pre- and post-meditation training doubtful.

However, leaving these concerns aside, we are still faced with the observation that all three studies investigating mindful breath awareness practice did not yield clear cut effects on self-reported mindfulness or affectivity. In the current study only a marginally significant increase in negative affect was observed in the meditation group. These results are in line with conceptualisations of mindfulness meditation which assert that attentional functions are developed first and constitute the basis for the subsequent cultivation of a non-reactive and non-judgmental mental state^9,61,62^. Given that we focused on cognitive processes and did not include robust behavioural or neuroscientific measures of affectivity or emotion regulation, we cannot draw strong conclusions on this matter. We may however, speculate that within the 8-week period of relatively modest simple mindful breath awareness meditation cognitive processes have improved, whereas the refinement of emotion regulation processes may require more time to develop^21^. This could mean that the primary role of this type of meditation within standard MBIs is to create the cognitive conditions for the affective and attitudinal changes to take hold. However, as there is hardly any research on the interplay between cognitive and affective processes related to mindfulness meditation such propositions are merely speculative.

In terms of potential therapeutic effects of mindfulness meditation it is important to emphasise that we investigated *one specific meditation practice*, rather than the typical complex mindfulness-based intervention. While the study clearly demonstrated cognitive improvements, these effects should not be misconstrued as therapeutic effects and should by no means be taken as evidence for therapeutic effectiveness. Thus, a conclusion that 8 weeks of mindful breath awareness meditation would lead to clinically or therapeutically relevant improvements is not warranted and would misrepresent our results. In conclusion, this study provides the first electrophysiological and behavioural evidence of efficiency gains resulting from meditation in a demanding cognitive task that combines selective attention, executive control and visual short-term memory, by means of a randomised active control group study. It highlights the potential of using the scalable MOT task for tracking performance improvements and the SSVEP paradigm for investigating neural efficiency gains related to meditation practice.

## Methods

### Participants

Forty-one participants without any previous experience in meditation and relaxation techniques or related exercises participated in the study in exchange for course credit or monetary reward. All participants confirmed that they had no history of drug abuse or video gaming experience^43,63^.

Data from seven participants were excluded from analysis. Four were excluded after the first measurement due to excessive artefacts of the EEG data and three because they did not regularly participate in the training (two from the PMR and one from the MED group). The remaining 34 participants were randomly allocated to the meditation and relaxation group (MED: N = 17; mean age = 20.82, SD = 2.01, 13 females, 12 right-handed; PMR: N = 17; mean age 21.47, SD = 5.03, 13 females, 15 right-handed).

### Procedure and design

Participants were recruited via the University’s email list with detailed information about the study, but no further information about the hypotheses. This research study was completed in accordance with the Helsinki Declaration and the guidelines of the ethics committee of Osnabrück University, who provided general approval for human SSVEP-EEG, a non-invasive standard procedure in our lab. All participants gave their informed written consent.

After the first testing session, participants were randomly (using the Matlab “rand” function) assigned to either the meditation group or the progressive muscle relaxation group. Statistical analysis confirmed that there were no significant between-group differences regarding age, gender, and handedness (all p > 0.2). Over a period of 8 weeks, both groups met for six 1.5-hour sessions that were guided by instructors with more than ten years of experience in guiding meditation or PMR sessions, respectively. A minimum of one home practice session per week and a maximum of one missed group session were pre-requisites for inclusion in the second measurement. All 34 participants included in the final analysis met these criteria.

### Relaxation training

The progressive muscle relaxation group engaged with a relaxation protocol based on Jacobsen (1938), which involves the deliberate build-up of tension and relaxation of various muscle groups following pre-defined sequences. The focus is on the perceived difference between these two physiological states resulting in an improved physical awareness and a general decrease of muscle tension and stress. The premise of this technique is that stress induces muscle tension (see e.g.^64^) and that, in turn, a systematic reduction of muscle tension should reduce psychological stress^65^. Specifically, it has been shown that PRM reduces cognitive, somatic, general state anxiety and somatic stress^66^.

### Meditation training

The meditation group was introduced to simple mindful breath awareness meditation that engages the key components of mindfulness meditation^25^. It entails directing the focus of attention to the air flow at the nasal tip or to the movement of the abdomen when naturally in- and exhaling, without applying any force or manipulating the natural breathing rhythm. Whenever the attentional focus slips away from the sensation of the breath and the mind wanders off, participants were instructed to become aware of the distraction and to redirect the attention back to the somatosensory experience of breathing. Participants were additionally coached to establish and maintain a non-judging, non-evaluative and accepting mental stance regarding their own mind-wandering and all other arising thoughts, feelings, sounds and sensations that attracted their attention. The refinement of the attentional focus combined with a non-elaborating, but curious, open and unbiased attitude towards mental experiences that acknowledges their presence without further engagement or suppression was revisited and emphasised throughout the course. This mindful breath awareness meditation can be understood as a combination focused attention (FA) meditation and open monitoring (OM) meditation^9^, with a stronger emphasis on FA in individuals who only start engaging with meditation, as was the case in this study^25^. Dahl *et al*.^57^ focus on cognitive processes in their classification of meditation practices. Within their framework this meditation would be classed as belonging to the “attentional family” of meditations, aiming to strengthen self-regulation of attentional processes, especially the ability to sustain meta-awareness. In line with these perspectives, the training incorporates the key components of mindfulness meditation the field appears to broadly agree upon, namely attention and awareness, qualified by an open, curious, accepting and non-judging attitude^15,24,67,68^. The same meditation approach had been successfully used in previous studies^29,60^.

For tracking the meditation or PMR progress and to encourage self-reliant mindfulness/PMR training, participants were handed a diary. In this diary, participants noted weekly on how many occasions they meditated/performed PMR, how long each session lasted, at what time they practiced as well as further comments. It was emphasised that the data would be processed anonymously and the participant’s honesty when reporting their progress would be crucial to the success of the study. They were assured that they would receive full course credit/compensation for the study, even if they do not practice at all.

### Questionnaires

For pre- and post-measurements, both groups completed two questionnaires right before the MOT task. To assess current affect we applied the Positive and Negative Affect Schedule (PANAS/state; 10 positive and negative items on a five-point Likert scale from ‘I don’t feel like this at all’ to ‘… highly’, Cronbach’s α > 0.84^69^; validated German version^70^. As a measure of mindfulness we applied the validated German version^71^ of the Five Facet Mindfulness Questionnaire^72,73^. The FFMQ comprises the subscales: (1) Non-reactivity to Inner Experience, (2) Observing/noticing/attending to sensations/perceptions/thoughts/feelings, (3) Acting with awareness/automatic pilot/concentration/non-distraction, (4) Describing/labelling with words and (5) Non-judging of experience. The FFMQ has received generally positive feedback from researchers, its internal consistency, as indexed by Cronbach's α, ranges from 0.67 to 0.93 for the subscales^74^.

### Multiple Objects Tracking Task

**Figure 2:**
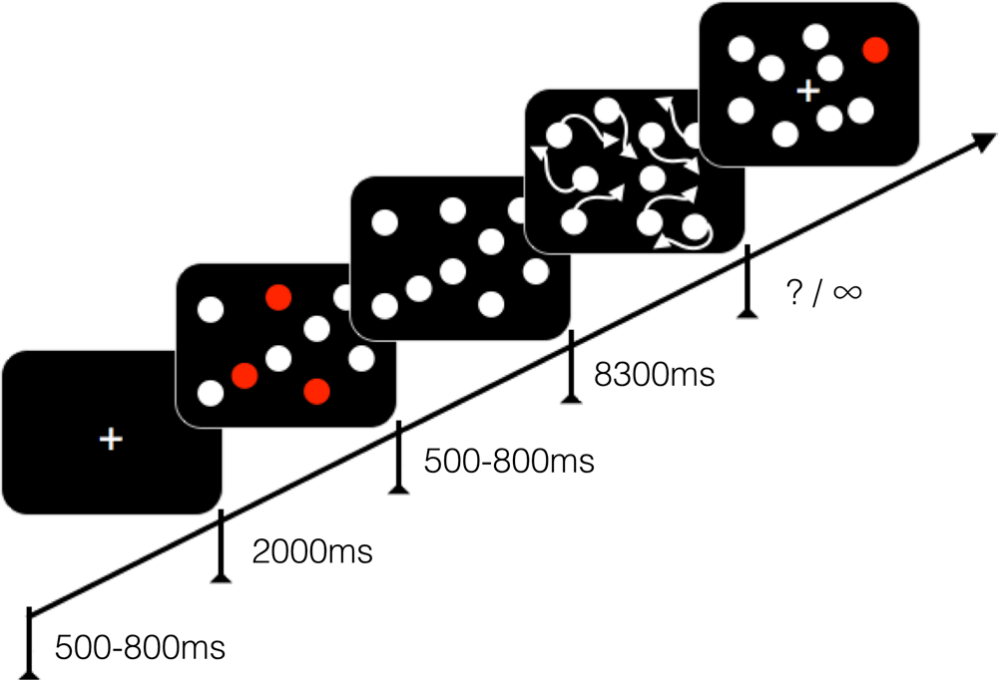
Schematic depiction of the multiple object tracking task. Screen size, relative disk size and number of disks are not to scale. See detailed description of the task in the text.

Each trial started with a black screen, displayed for 500–800 ms followed by the display of 16 randomly placed identical white disks and a fixation cross in the middle of the screen. To indicate which disks need to be tracked throughout a particular trial, depending on the condition, two, three, four or five disks turned red or green for two seconds. The colour for labelling the targets was counterbalanced across groups and participants. Following this, all disks turned white again and remained static for 500–800 ms, before starting to move for 8300 ms. Each disk moved at a speed of 1.4°/s on a linear path for a distance of 0.7° to 1.2° and then changed their direction by either 22.5° or 44.5°. The disks never “collided” or overlapped. When encountering a border of the rectangular presentation area (7.15° horizontal × 5.25° vertical), a disk would “bounce off”. While moving, all disks flickered at a frequency of 11Hz. Participants were instructed to track all disks that were highlighted at the beginning of the trial while maintaining constant gaze on the fixation cross. At the end of each trial, one of the 16 disks was highlighted in the target colour, and the participant was required to indicate whether it was one of the tracked disks or not. In half of the trials a target disk was highlighted. The experiment comprised 4 blocks with 40 trials each, with 10 trials per condition. Between blocks, participants took short breaks (Fig. 2).

### Electroencephalographic recording and data analysis

**Figure 3:**
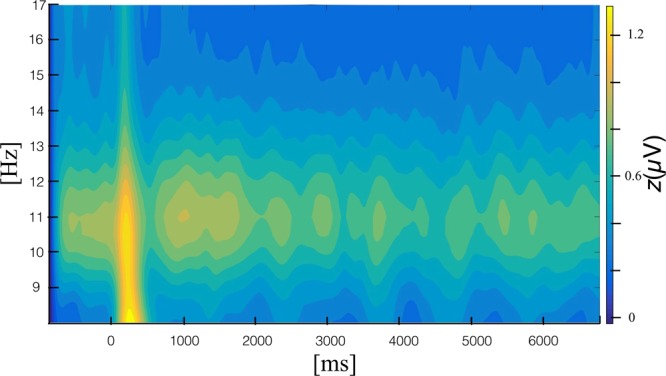
Time-frequency plot, exhibiting the steady-state visually evoked potential at 11 Hz averaged over all sensors.

EEG was continuously recorded using 128 Ag/AgCl electrodes on an Active-Two amplifier system (BioSemi, Amsterdam, Netherlands) with a sampling rate of 512 Hz. To monitor blinks, we recorded the electro-oculogram. Two additional electrodes were used as reference and ground electrodes (CMS and DRL; cf. http://www.biosemi.com/). EEG was segmented to obtain epochs starting 600 ms before to 7000 ms after movement onset. Artefact correction was performed using statistical correction of artefacts in dense array studies (SCADS)^75^. Epochs containing excessive ocular artefacts or more than 20 artefact-contaminated channels were automatically excluded from further analysis. If 20 or less spatially distributed channels contained noise, these were replaced by interpolation. In total, approximately 30% of all epochs were rejected. Each epoch was detrended and no further filters were applied. For subsequent analyses, we used the average reference. To determine the changing magnitude of the steady state visually evoked potential, the event-related response was spectrally decomposed using Morlet wavelet analysis (approximately 12 cycles per wavelet, averaged wavelet duration approximately 182 ms, averaged spectral bandwidth approximately 1.75 Hz^76^ Fig. 3).

**Figure 4:**
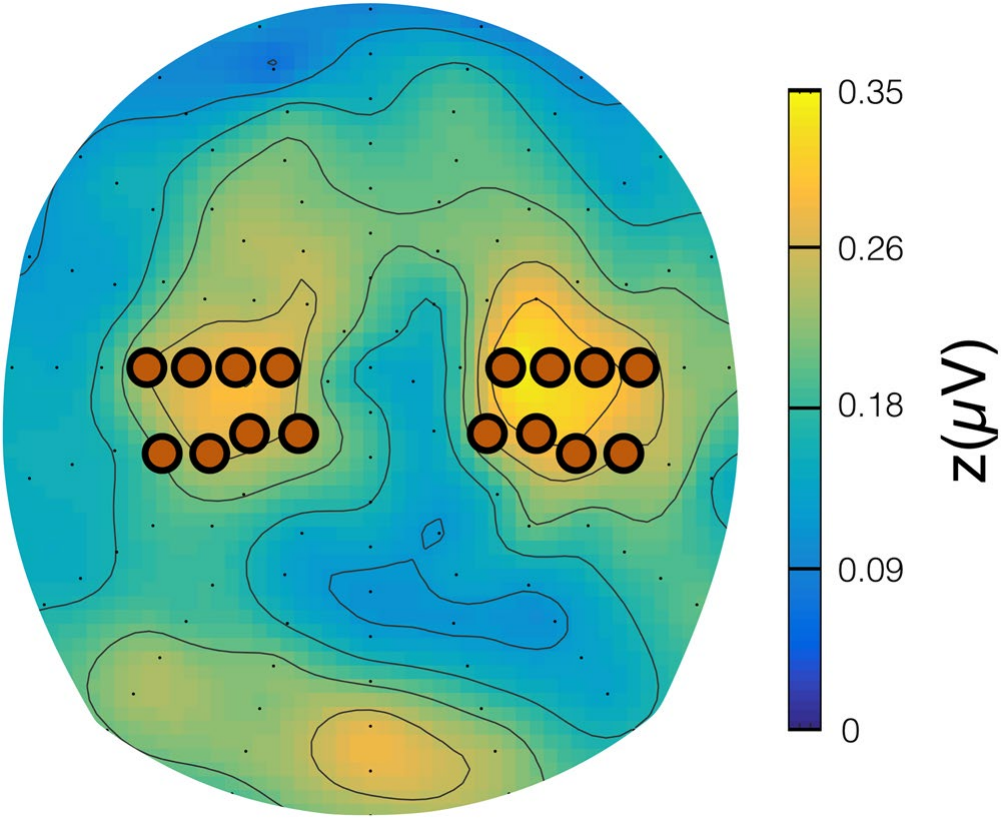
Spherical spline-interpolated topography of the grand mean SSVEP amplitudes, averaged over factors GROUP, CONDITION and TIME from 500 ms to 6800 ms post flicker-onset. SSVEP maxima over left and right hemispheres were identified at temporoparietal sensor sites in vicinity of standard electrode positions C3 and C4, respectively. The SSVEP signals at the highlighted electrodes were subjected to further analysis.

**Figure 5:**
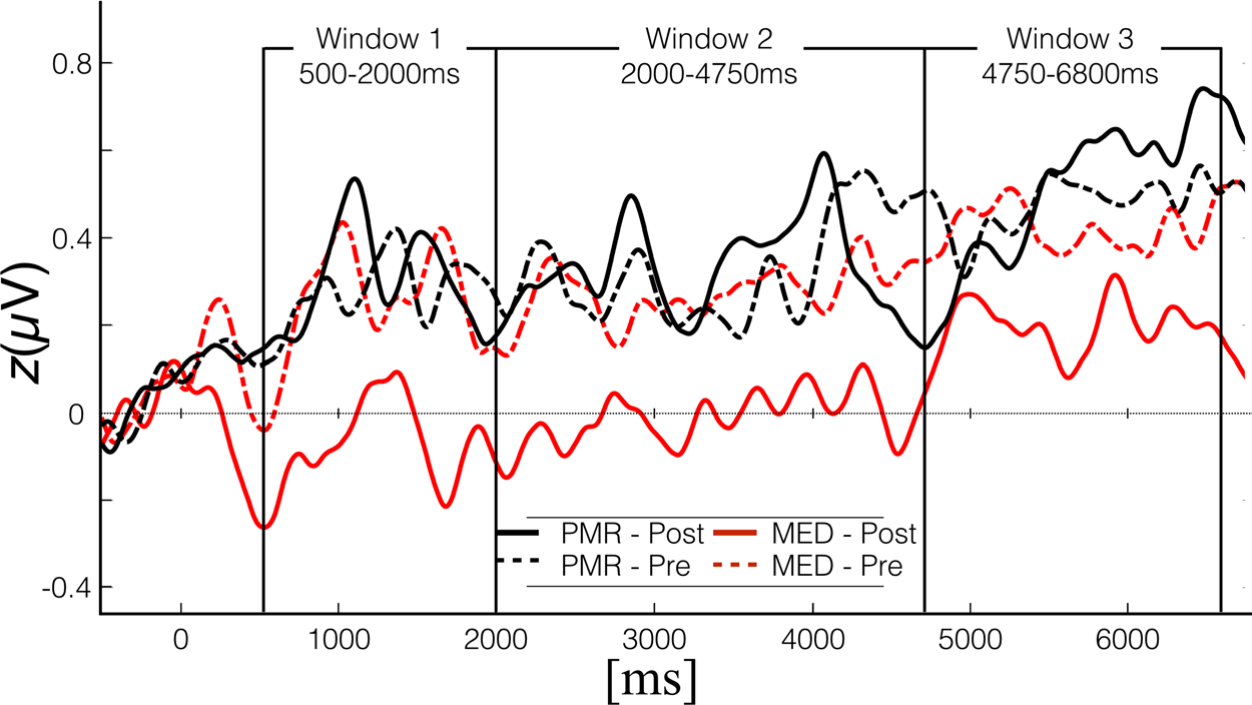
Time amplitude plot of z-transformed waveforms at chosen sensor sites for both groups before and after their respective training.

**Figure 6:**
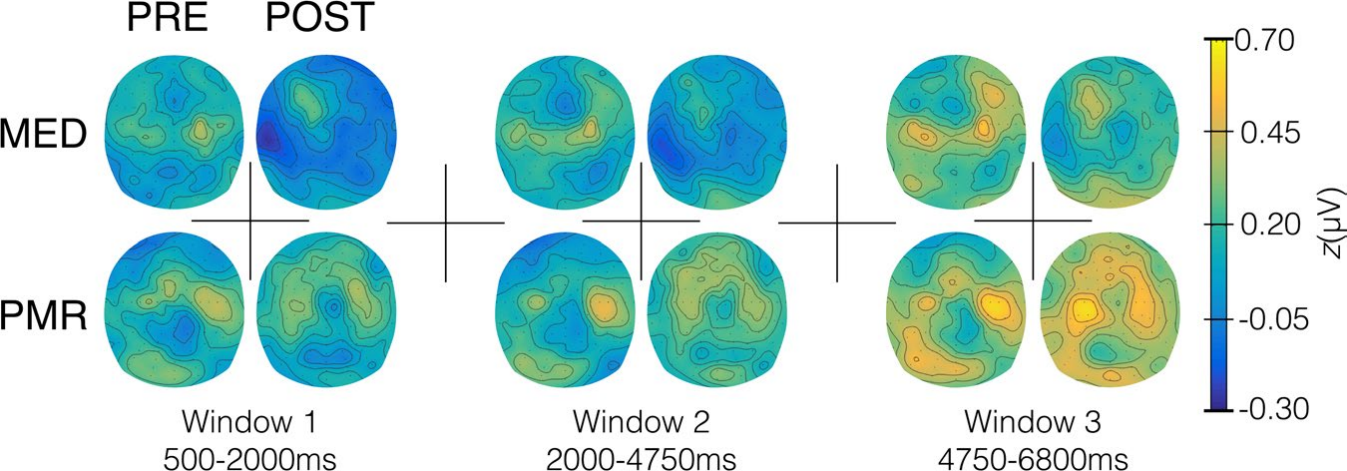
Spherical spline-interpolated topographies for the three consecutive time windows for the MED and PMR group before and after their respective training. Widespread lower amplitudes are clearly visible after meditation but not after progressive muscle relaxation.

The period of 500 ms to 100 ms before stimulus onset served as baseline and its mean amplitude was subtracted from each epoch. For analysing SSVEP the electrodes with the highest signal averaged over all conditions were determined by visual inspection of the grand mean topography (Fig. 4). A time window from 500 ms to 6800 ms was determined and for the explorative investigation of temporal dynamics further subdivided into three distinct time segments (T1: 500–2000 ms, T2: 2000–4750 ms and T3: 4750–6800 ms) by visual inspection of the time-by-amplitude plots at these electrodes (Figs 5 and 6). For statistical analyses mean amplitudes were calculated over selected time windows and electrodes.

### Statistical Analysis

Prior to further analysis, electrophysiological data were z-transformed across time samples, conditions, and groups separately for each participant, each electrode, and each frequency (see^51^). All data were subjected to 2 × 2 × 4 repeated measurement ANOVAs with factors GROUP (MED, PMR), TIME (PRE, POST) and CONDITION (No. of disks; 2, 3, 4, 5) followed by post-hoc *t*-test to probe significant interactions. ANOVAs for the SSVEP data were calculated for the whole analysis period (500 ms to 6800 ms) and also separately for each of the identified time segments. Self-report data were analysed with 2 × 2 (GROUP × TIME) mixed ANOVAs, followed by post-hoc t-tests to probe significant interactions, for PANAS-PA, PANAS-NA and the FFMQ total score. Potential effects on the five mindfulness facets of the FFMQ were explored by subjecting each FFMQ-subscale to the same 2 × 2 ANOVAs. If sphericity assumptions were violated, the Greenhouse-Geisser corrected *F* values are reported.

## Data Availability

The datasets generated and analysed during the current study are available from the corresponding author on reasonable request.
